# Effect of Scanning Strategy on the Manufacturing Quality and Performance of Printed 316L Stainless Steel Using SLM Process

**DOI:** 10.3390/ma17051189

**Published:** 2024-03-04

**Authors:** Zhijun Zheng, Bing Sun, Lingyan Mao

**Affiliations:** School of Mechanical & Automotive Engineering, South China University of Technology, Guangzhou 510641, China; yasunbing@163.com (B.S.); congcong_yan@126.com (L.M.)

**Keywords:** selective laser melting, scanning strategy, electrochemical tests, mechanical properties

## Abstract

In this study, the effects of Z-0°, Z-67°, Z-90°, I-67°, and S-67° scanning strategies on the surface morphology, microstructure, and corrosion resistance of the specimens in SLM316L were systematically studied. The results show that the partition scanning path can effectively improve the manufacturing quality of the specimen, reduce the cumulative roughness layer by layer, and increase the density of the specimen. The scan path of the island partition of the fine partition is better than that of the strip partition; moreover, the 67° rotation between each layer reduces the accumulation of the height difference of the melt pool, fills the scanning gap of the previous layer, and improves the molding quality of the sample. Electrochemical tests were performed in an aqueous solution of NaCl (3.5 wt%), including open-circuit potential (OCP), dynamic potential polarization, and electrochemical impedance spectroscopy (EIS). The results show that the specimen with a 67° rotation between each layer achieves stability of the surface potential in a short time, and the I-67° specimen exhibits good corrosion performance, while the Z-0° specimen has the worst corrosion resistance.

## 1. Introduction

Additive manufacturing (AM) has the advantages of short development cycle and personalized customization. It is developing rapidly for use in the formation of metal parts [1,2]. Selective laser melting (SLM) is a widely used additive manufacturing technique for metals. This method uses a laser spot with a small spot diameter, which has the characteristics of high manufacturing accuracy and high density and can achieve a one-time net shape [3,4].

In the past decade, the research of SLM technology has mainly focused on the optimization of laser energy parameters and mechanical properties [5,6]. It is generally believed that laser energy density plays an important role in densification. There is a positive correlation between laser energy density and densification and then a negative correlation [7,8].

At the same time, the laser energy density also has a significant effect on the roughness. As the energy density increases, the width of the melt pool expands, reducing the top surface roughness. The increase in energy density also increases the side temperature, resulting in severe stickiness and improved side roughness [9]. In the study of mechanical properties, the metal materials prepared via SLM technology have a fast solidification speed. The metal materials formed have a small grain size, fine sub-grain structure, and high dislocation density at grain boundaries. Due to the effects of grain refinement and dislocation, the samples prepared by SLM are significantly better than the forged samples [10]. In the samples prepared by SLM, the samples in different orientations exhibited significant mechanical anisotropy. When the melt pool boundary (laser scanning direct area) is parallel to the loading direction or angle of the force, the anti-sliding deformation ability of the melt pool is large [11]. Therefore, when the specimen is loaded perpendicular to the construction direction, the specimen exhibits better mechanical properties [12].

At present, a large number of studies have focused on the influence of process parameters on SLM [13,14,15,16,17], and there are few studies on the scanning strategy of microstructure and corrosion properties; these studies mainly focus on the optimization of densification and manufacturing accuracy. Li et al. [18] studied the effect of the scanning strategy on the pores of AlSi10Mg alloy, and the results showed that alternating interlayer and laser remelting could effectively improve the sample density and make the sample pore distribution uniform. El et al. [19] improved corrosion resistance under acidic conditions by coating the surface of 3D-printed H13 steel. Zhang et al. [20] studied the influence of scanning strategy on the molding accuracy of the specimen and found that a certain rotation angle between layers can effectively reduce the roughness degree of the top surface. Ge et al. [21] found that during the printing process, each layer was rotated by 67°, and the prepared samples had good molding quality and mechanical properties. In conclusion, the scanning strategy does have a deep effect on the metal materials prepared by SLM. Therefore, it is necessary to explore the influence of scanning strategy on the manufacturing quality and corrosion performance of metal materials prepared by SLM.

In this paper, 316L stainless steel materials prepared by SLM were taken as the research object. Firstly, the morphology and roughness of the original sample were characterized via scanning electron microscope and three-dimensional profilometer, then the microstructure of the sample was characterized via OM and XRD; finally, the corrosion performance of the sample was characterized via electrochemical experiments, and the influence of scanning strategy on molding quality and corrosion performance was studied.

## 2. Materials and Methods

**Figure 1:**
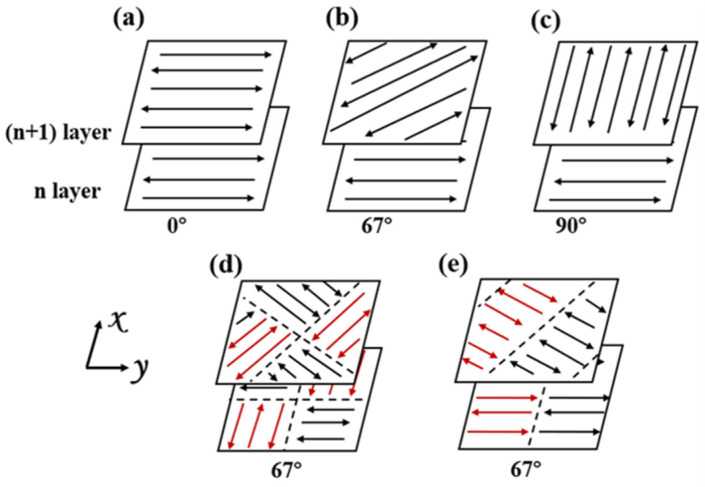
Schematic diagram of scanning strategy: (**a**) Z-0°; (**b**) Z-67°; (**c**) Z-90°; (**d**) I-67°; (**e**) S-67°.

Gas-atomized spherical 316L ASS powder (15~53 microns, provided by AviMetal Powder Metallurgy Technology Co., Ltd., Beijing, China) with the nominal composition listed in Table 1 was used in this study. A block of 316L ASS with the size of 20 mm (width) × 20 mm (length) × 10 mm (thickness) was fabricated by SLM (Laseradd, Guangzhou, China). During the SLM process, the layer thickness, laser power, hatch spacing, and scan speed were set as 30 μm, 165 W, 70 μm, and 800 mm/s, respectively. These parameters are the standard parameters set by the machine manufacturer for obtaining samples with near full density. Five different scanning strategies were used for sample preparation, as shown in Figure 1. In Figure 1a–c, the same scan path, the Z-scan path, is used. The rotation angles between layers are different, with rotations of 0°, 67°, and 90°, respectively. Figure 1d uses an islanded scanning path to divide the scanning area into a square with a width of 7 mm and a 67° rotation between layers. Figure 1e shows a strip scanning path with a 7 mm width and 67° rotation between layers.

**Figure 2:**
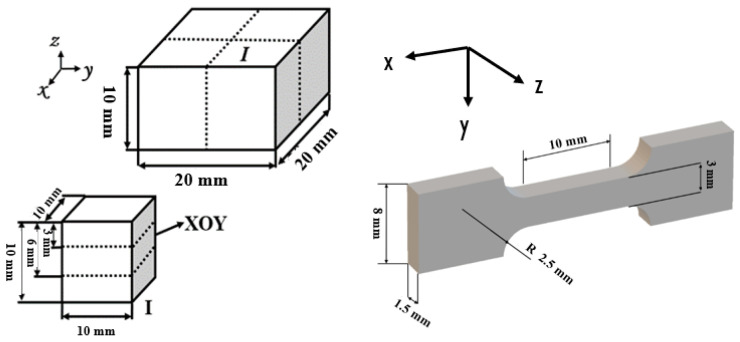
Sample schematic diagram of cutting.

After printing, the top surface topography of the sample was observed using an S-3700N tungsten scanning electron microscope, and then the roughness of the top surface of the sample was characterized by a VR-600 series 3D profile meter. The cutting diagram of the sample is shown in Figure 2. The printed samples include some cubs with the gauge dimensions of 20 mm (length) × 20 mm (width) ×10 mm (height), and some flat, dog-bone-shaped tensile samples with gauge dimensions of 3 mm (width) × 10 mm (length) × 1.5 mm (thickness). The cuboid is furtherly divided into four 10 mm × 10 mm × 10 mm cubes. The intermediate samples of region I are taken as XOY samples, respectively, with a sample size of 10 mm × 10 mm × 3 mm. The rough surface of the sample was polished with 200~2000 mesh sandpaper, and the bulk density of the sample was calculated by the Archimedes drainage method [22]. The phase structure of the sample was analyzed using a Netherlands PHILIPS X’pert MPD Pro X-ray diffraction (XRD), Eindhoven, Netherlands. The acceleration voltage was 45 kV, the acceleration current was 45 mA, the scanning speed was 2°/min, the scanning step was 0.02°, and the scanning angle was 30~90°. Etching solutions were prepared with HF, HNO_3_, and H_2_O at a volume ratio of 2:1:7. The sample was placed in the etching solution for about 60 s for chemical etching. The microstructure of the samples was characterized using a German Leica DMI 3000M light microscope, Wetzlar, Germany. The tensile performance test equipment is an RGL-20A microcomputer-controlled electronic universal testing machine, and the crosshead displacement speed is 1 mm/min until the sample breaks and the yield strength, tensile strength, and elongation of the sample are obtained.

Electrochemical testing is used to characterize the corrosion properties of a sample. Pitting corrosion is one of the important failure forms of stainless steel, and Cl^−^ is a typical pitting corrosion-inducing factor for stainless steel [23,24,25,26]. The electrochemical test was performed in a 3.5% sodium chloride solution at 25 °C. The test equipment is a Gamry reference 3000 potentiostat using a conventional three-electrode battery system. The reference electrode is Ag/AgCl, the counter electrode is a graphite rod, and the working electrode is the sample to be measured. Prior to the electrochemical experiment, cathodic polarization was performed at a voltage of −1.5 V for 300 s to remove the thin film formed in the air on the surface of the sample. Before measuring the open-circuit potential (OCP) of the sample, the sample is soaked in an electrochemical solution for 15 min and then tested for OCP. When the rate of change in the surface potential of the sample is less than 0.1 mv/s, the current potential is taken as the OCP value of the sample. Electrochemical impedance spectroscopy (EIS) is performed at an open-circuit potential. The impedance frequency is 10^−2^~10^5^ Hz, and the voltage amplitude is 10 mV. The measurement range of the dynamic potential polarization curve is −0.6~1.2 V(Ag/AgCl), and the scanning speed is 1.67 mv/s. All tests were repeated three times to ensure that the results were authentic and reliable.

## 3. Results and Discussion

### 3.1. Surface Topography and Roughness Analysis

**Figure 3:**
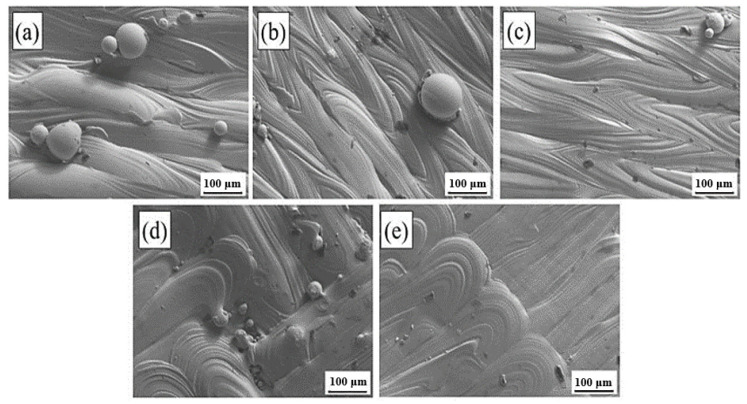
Surface morphology of samples printed with different scanning strategies: (**a**) Z-0°; (**b**) Z-67°; (**c**) Z-90°; (**d**) I-67°; (**e**) S-67°.

Figure 3 shows the surface topography of a 316L stainless steel sample prepared using different scanning strategies. Figure 3a–c uses the Z-shaped scanning path with 0°, 67°, and 90° interlayer rotation, respectively, whereas Figure 3d,e uses 67° interlayer rotation with island and bar scanning paths, respectively.

It can be clearly seen that compared with the samples with 67° and 90° interlayer rotation, there is a certain height difference in the molten pool with 0° rotation between the layers. The melt pool center is significantly higher than the melt pool center on both sides of the molten pool; the island, and STR IP scanning paths are both partitioned by the method of partition scanning, and the overlapping areas of the separator form a good metallurgical combination.

**Figure 4:**
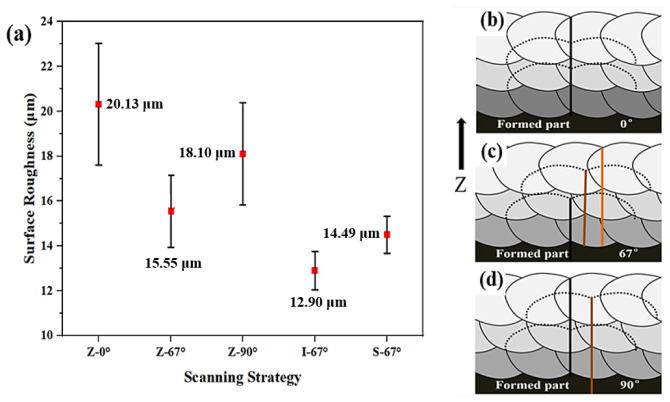
(**a**) Analysis of the surface roughness of the sample using different scanning strategies; sample pool fittings with different rotation angles: (**b**) 0°; (**c**) 67°; (**d**) 90°.

Figure 4a shows the effect of different scanning strategies on the surface roughness of the sample. Among the 316L stainless steels prepared by a Z-shaped scanning path, the top surface roughness is the lowest when the interlayer rotation is 67°, and the top surface roughness is the highest when the interlayer rotation is 0°. This phenomenon is mainly affected by the phenomenon of the height difference between the center of the melt pool scan line and the surrounding height accumulating layer by layer. When the laser path of the current two layers is the same, the difference between the height and the low degree formed by the laser scan of the previous layer cannot be compensated for in the next layer, and the layers accumulate to form a rougher upper surface. As shown in Figure 4b, the lowest point of each layer of the melt pool coincides in the *Z*-axis direction, and the lowest part of the melt pool of the n, (n + 1), and (n + 2) layers are on the same straight line as the highest point of the melt pool (the black line in Figure 4b is the lowest point of the melt pool overlap, the yellow and brown lines indicate that the nadir will be covered by other layers of melt pools), and the cumulative height difference of these layers causes the surface of the sample to be rough.

**Figure 5:**
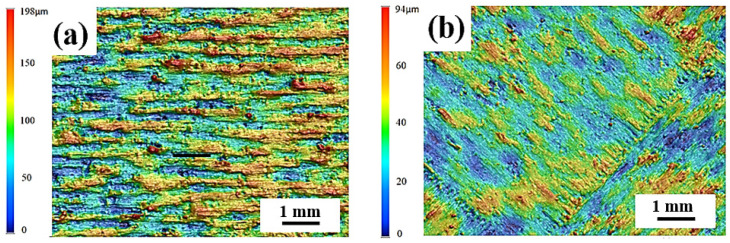
The surface roughness of the printed part captured at Z-0° (**a**) and I-67° (**b**) angles.

For the 316L stainless steel specimen prepared by the Z-scan path, the roughness of the top surface of the specimen with 67° rotation between each layer is 90° lower than that between the layers, which is also affected by the height difference between the center of the melt pool and the surrounding layers (Figure 4c,d). Moreover, the melt pool (67°) between the t, n, (n + 1), and (n + 2) layers does not coincide along the *Z*-axis; thus, the formation of the molten pool of the next layer can make up for the height difference and improve the molding quality of the top surface. For samples with 90° rotation between layers, the lowest melt pools of the nth and (n + 1) layers do not coincide in the *Z*-axis direction, but the lowest points of the melt pools of the (n + 2) and nth layers coincide; thus, there is still an accumulation of melt pool height differences. Finally, the Z-67° sample has the lowest surface roughness. I-67° and S-67° use islanding and bar scanning paths, with a short single laser path and low heat accumulation along the *Z*-axis, which is more conducive to the lower melt channel to absorb heat from the upper melt channel and slow down the roughness accumulation along the *Z*-axis, whereas the I-67° scanning strategy has a finer partition, lower heat accumulation along the *Z*-axis, and the lowest sample surface roughness [16,27,28]. Figure 5 provides a clearer comparison of the difference in surface roughness between Z-0° and I-67°.

### 3.2. Density Analysis

**Figure 6:**
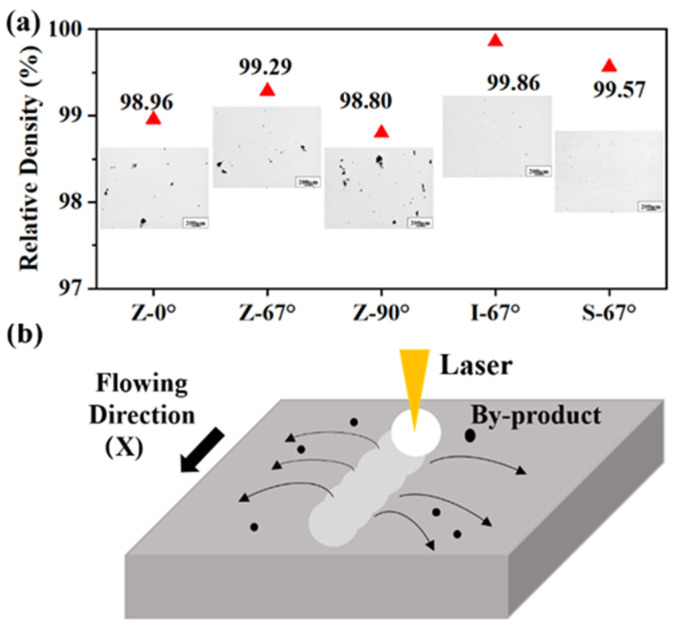
(**a**) Relative density of samples under different scanning strategies; (**b**) schematic diagram of the scanning direction parallel to the direction of the shielding gas flow.

The density of the sample was measured using the Archimedes drainage method, and the results are shown in Figure 6a. As can be seen in Figure 6a, the bulk density of the sample is above 98%. Among them, the lowest density of the Z-90° specimen is 98.80%, and the highest density of the I-67° specimen is 99.86%.

In the Z-scan path, the sample prepared by rotating 67° between the layers has the highest density for the same reason as the roughness of the sample. Limited by the molding characteristics of laser selective melting technology, it is easy to generate unmelted powder pores and pores at the interlayer joint. Using the same interlayer laser path at 0° interlayer rotation, the n-layer laser scans the powder that has not yet melted and may not be completely melted at the (n + 1) layer, manufacturing defects to reduce the density of the sample. The interlayer rotation angle can effectively improve the meltability of the interlayer powder and increase the bulk density of the sample. However, the molar mass of the sample is also affected by the protective airflow and printing by-products (ejection powder and spatter) [29,30]. As shown in Figure 6b, when the interlayer rotation is 90°, a laser path parallel to the direction of the protective airflow will appear in every other layer, and the molding quality will be affected by the laser energy attenuation and by-product spatter. Splashes and welding fumes from the process area are not easily removed sufficiently, resulting in increased interaction between the laser and process by-products, with the consequence of the attenuation of the laser spot [31]. The Z-shaped scanning path is long, and when the laser path is parallel to the airflow, some studies have pointed out that the laser energy attenuation reaches its maximum at this time, reducing the utilization rate of the laser [32]. In addition, the scan line formed by laser scanning is parallel to the direction of the airflow, and the splash direction of the by-product is perpendicular to the direction of the purging; the direction of the purging makes it more difficult for the by-product to be blown away from the sample surface, reducing the sample density while increasing the roughness of the sample surface [33].

When using islanding and strip scanning paths, the partition scanning method is adopted; the single laser scanning path is short, the by-product generation density is low, and the laser utilization rate is high. Finally, the impact of scanning by-products is small, which greatly improves the quality of sample molding. As a result, the density of the I-67° and S-67° samples is higher than 99.5%.

### 3.3. Microstructure and Phase Analysis

**Figure 7:**
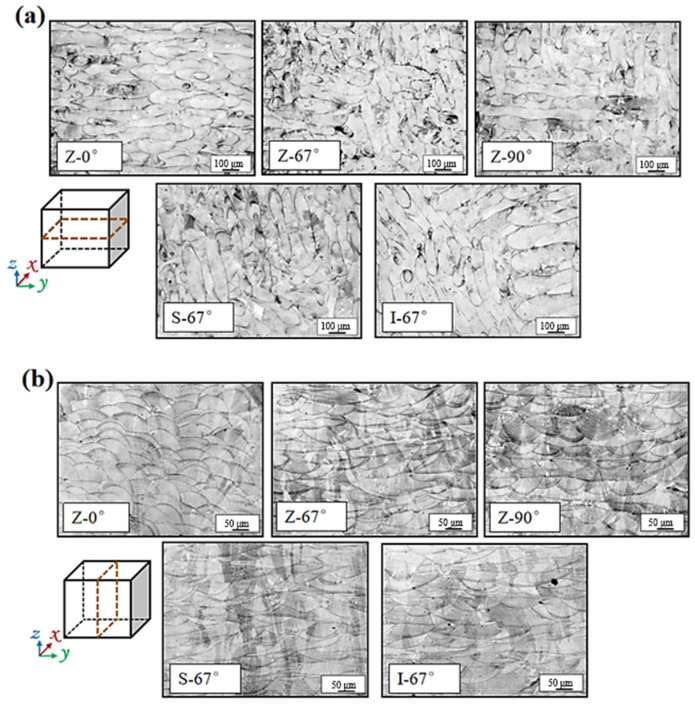
(**a**) Optical micrograph of the XOY plane; (**b**) XOZ plane of the sample with different scanning strategies.

Figure 7 shows the metallographic diagram of a 316L stainless steel sample prepared by SLM under different scanning strategies. As can be seen from Figure 7a, the top surface (XOY) metallographic is composed of a number of scan lines. The scan lines of Z-0° are parallel to each other, and the scan lines of Z-90° samples are perpendicular to each other in two directions. The other three samples are rotated 67° between the layers, and the scan line is more oriented in the XOY plane. Figure 7b shows the side (XOZ) metallographic of the specimen, which is mainly composed of a scan line and the side of the fish scale melt pool. The metallographic phase on the side of the Z-0° specimen is composed of a fish-scale molten pool. On one side of the Z-67° sample and the I-67° sample, the scale melt pool and the strip melt pool intersect, and the scan line layer appears after about two layers of melt pool structure. The Z-90° specimen has the same laser path in every other layer; the fish scale pool and the strip pool alternate, and the scan line appears after the appearance of one layer of the scale pool.

**Figure 8:**
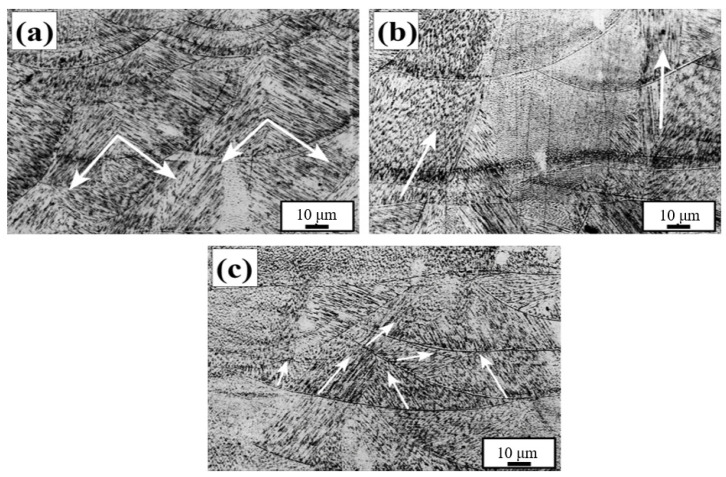
Comparison of microstructure between samples with different scanning strategies: (**a**) Z-0°; (**b**) Z-67°; (**c**) Z-90°.

Figure 8 shows transverse metallographic magnifications of Z-0°, Z-67°, and Z-90° samples. The laser selective melting process uses high-energy lasers to melt metal powder in layers to achieve the purpose of personalized molding of metal parts. This metal formation process is characterized by high laser energy and fast movement speed, resulting in a large temperature gradient and a large growth rate, manufacturing a very large cooling rate (cooling rate = G × R) in the molten pool range and manufacturing a fine cell-like crystal structure in the solidified grains [34]. This fine cell-like crystal structure is not confined to a single pool boundary, and some grains are transpelled through the pool boundary for epitaxial growth. The same laser energy density and laser path were used for the three different samples, but there were significant differences in the transverse metallographic morphology, mainly due to the different temperature gradients and interlayer rotations of the samples.

Grain growth is mainly carried out in the direction of the maximum temperature gradient. The maximum temperature gradient direction of the Z-0° sample points to the center of the melt pool [35]. That is, the direction is perpendicular to the melt pool boundary, where there are two different temperature gradients in the melt pool, and the grain growth direction is shown by the white arrows in the figure, growing in two directions perpendicular to each other, depending on the maximum temperature gradient. In Figure 8b, when the interlayer rotation is 90°, the maximum temperature gradient of the melt pool formed on one side of the scan line also points to the position of the central melt pool, that is, perpendicular to the boundary of the scan line, and coincides with the direction of the building (*Z*-axis). When the interlayer rotation is 67°, the angle difference between the scan lines of different layers is large, and after multi-layer scanning, the single-layer scanning vector direction of the laser coincides with the existing scan lines, resulting in a large temperature gradient gap between layers. The direction of the temperature gradient in the molten pool is also complex, and there are three different grain growth directions in the fish scale molten pool shown in Figure 8c.

**Figure 9:**
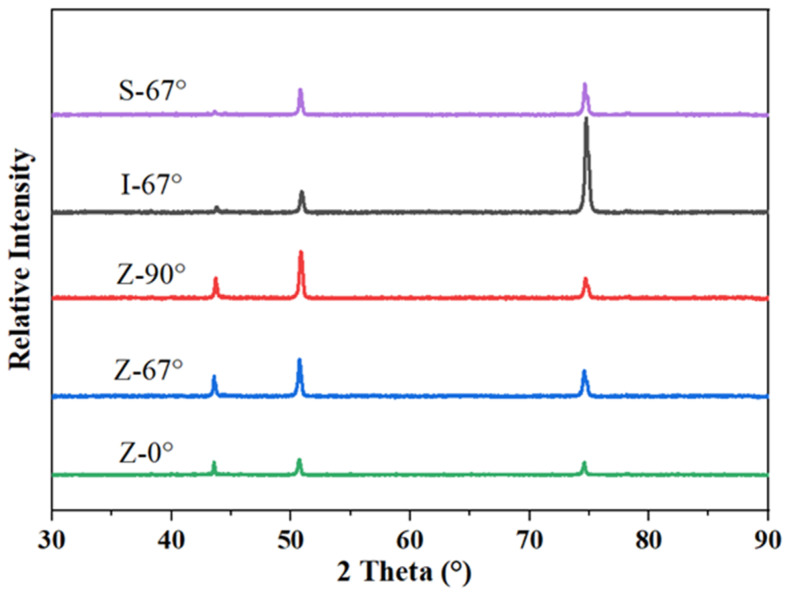
XRD profiles of samples with different scanning strategies.

As an austenitic stainless steel, 316L stainless steel is a face-centered cube (FCC) crystal structure, which is represented in XRD plots as three diffraction points: (111), (200), and (220). The XRD pattern of the experimental sample is shown in Figure 9, which shows the diffraction peak of the FCC crystal structure, indicating that the additive manufacturing technology did not alter the phase structure of the 316L stainless steel. Among the 316L stainless steel (Z-0°, Z-67°, Z-90°) prepared by the Z-scan path, the peak intensity of the (200) diffraction peak was the largest. When using the partition sweep path (I-67°, S-67°), the diffraction peak of (220) is the strongest, and the thinner the partition (220), the stronger the diffraction peak.

### 3.4. Corrosion Resistance

**Figure 10:**
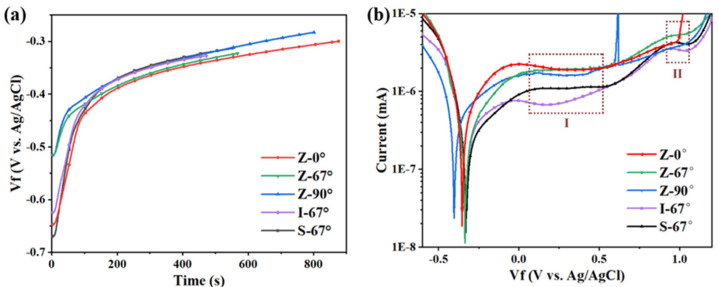

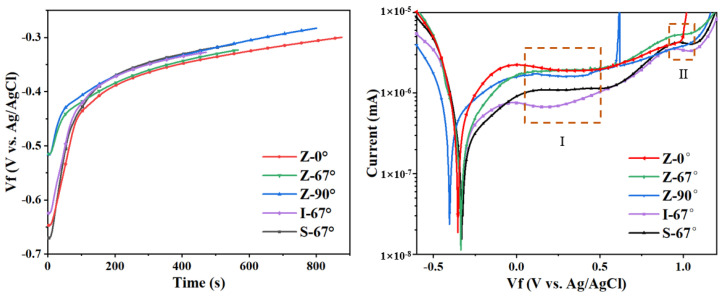
(**a**) OCP plot and (**b**) potentiometric polarization of the sample by different scanning strategies in NaCl solution.

Figure 10a is an OCP plot of the top sample with different scanning strategies, with a cathodic polarization of −1.5 V potential for 300 s to remove the passivation film formed in the air on the sample surface prior to OCP measurement. When the rate of change in the sample surface potential is less than 0.1 mV/s and lasts for more than 10 s, the potential at this time is the open-circuit potential of the sample surface. During the test, passivation films on the surface of different samples were formed at different rates, which resulted in different amounts of time taken for open-circuit potential punctures. As shown in Figure 10a, the I-67° sample first reaches a steady state, manufacturing a stable passivation film in the shortest possible time (464 s). The Z-0° sample reached a steady state at 871 s of the experiment, which took the longest amount of time. This difference is mainly influenced by two aspects: one is the effect of specimen defects on the passivation film formation rate [36], and the other is the different passivation film formation rates of different oriented grains [37,38].

Figure 10b shows the dynamic potential polarization curves of samples with different scanning strategies in 3.5% sodium chloride solution; the morphology and trend of the passivation films prepared by different scanning strategies are the same, and they can be explained in two stages. One is the self-corrosion potential before the cathodic polarization zone, and the other is after the self-corrosion potential in the anodic polarization zone; the difference in the corrosion behavior of the sample is mainly reflected in the anodic polarization zone. In the Z-scan path sample, the Z-90° sample experienced a large current increase and decrease at about 600 mV, indicating that the sample had metastable pitting corrosion. As the potential increases, the passivation film of the Z-67° and Z-90° samples does not break down under experimental conditions, and the passivation film of the Z-0° sample breaks down at about 1 V potential.

At the same angle of rotation between layers, two passivation zones appear in the anodizing regions of Z-67°, I-67°, and S-67°. The first passivation zone is the 50~500 mV region (recorded as the I zone). The current decreases slowly with the increase in potential in the early stage, and when the potential rises to about 300 mV, the current still increases slightly with the increase in potential; however, compared with the early anodic polarization, the current density increases relatively slowly, indicating that the passivation film is more stable in the potential region. When the potential of the I region is greater than the potential, the current increases with the increase in potential, and the increase in the current in the I region is more significant than that in the I region. The potential enters the range of 900 mV–1 V, and there is also a short passivation zone (recorded as the II zone). The passivation zone is short and soon enters the passivation stage, and the corrosion current density surges, indicating that under the action of potential anodic polarization, the dissolution rate of the passivation film increases, and the corrosion rate of the sample increases.

**Figure 11:**
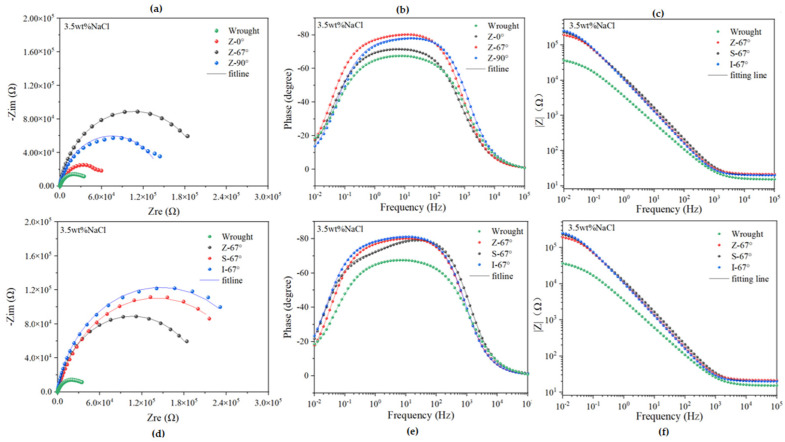
Nyquist impedance spectrum and Bode plots of the samples in 3.5 wt% NaCl: (**a**) Nyquist plots with different interlayer rotation angles; (**b**) Bode phase diagrams and (**c**) Bode impedance with different interlayer rotation angles; (**d**) Nyquist plots with different scanning paths; (**e**) Bode phase diagrams and (**f**) Bode impedance with different scanning paths.

As can be seen from the data in Figure 11a, the impedance diameter of the Z-67° specimen is significantly higher than that of the Z-90°, Z-0° and forgings under the Z-type laser path, indicating better corrosion resistance at a 67° interlayer rotation angle. In addition, the Bode plot of Figure 11b highlights that the time constant of the sample is twice as high at the intermediate frequency when the angle of rotation between the layers is different. As can be seen from the data in Figure 11d, the impedance diameter of the I-67° sample is significantly higher than that of the S-67°, Z-67°, and forged parts at a 67° angle of rotation between layers. It is shown that there is better corrosion resistance when using the islanded laser path. In addition, the Bode plot of Figure 11e highlights that the time constant of the sample is twice as high at the intermediate frequency when only the laser path is different.

**Figure 12:**
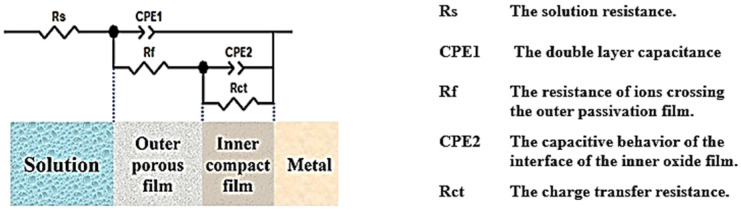
EIS equivalent circuit for SLM 316 stainless steel and its interpretation (in 3.5 wt% NaCl solution at 298 K).

Figure 12 shows the equivalent circuits of the Nyquist and Bode diagrams and the interpretation of the individual circuit elements in them. Among them, the protection behavior of electric double-layer capacitors is different from that of pure capacitors due to the effects caused by adsorption layer formation, pore defects, etc., which explains why CPE components and not capacitors are studied. The impedance of CPE is calculated by $Z={\left[Q\cdot {\left(\dot{J}\omega \right)}^{n}\right]}^{-1}$ [39] where *Q* is the admittance of the CPE in Ω·sn, s is the time unit second, j is the imaginary part, n is the exponent of the constant phase angular element, and *ω* is the angular frequency. The above formula can be used to calculate the capacitance value of a constant-phase angle element. Among them, the value of the constant phase angle element index n is taken as 0~1, CPE represents an ideal resistance if n = 0, and an ideal capacity if n = 1.

Table 2 lists the fitting results for equivalent circuits. According to the fitting results, Rs did not change much, indicating that the samples constructed under different scanning strategies had the same electrolyte behavior in solution. Corrosion resistance was assessed using the sum of Rf and Rct. In general, the higher their values, the better the corrosion protection. Of all the 316L stainless steels manufactured by SLM, the I-67° specimen has the largest sum of Rf and Rct, resulting in better corrosion protection. This is in good agreement with the results of the previous dynamic potential polarization curve test, which showed the best I-67° performance. This may be related to the reduction of pore defects, the smaller proportion of (111) faces, and the fewer ions diffusing near the matrix in samples at I-67° [40,41].

### 3.5. Mechanical Properties

**Figure 13:**
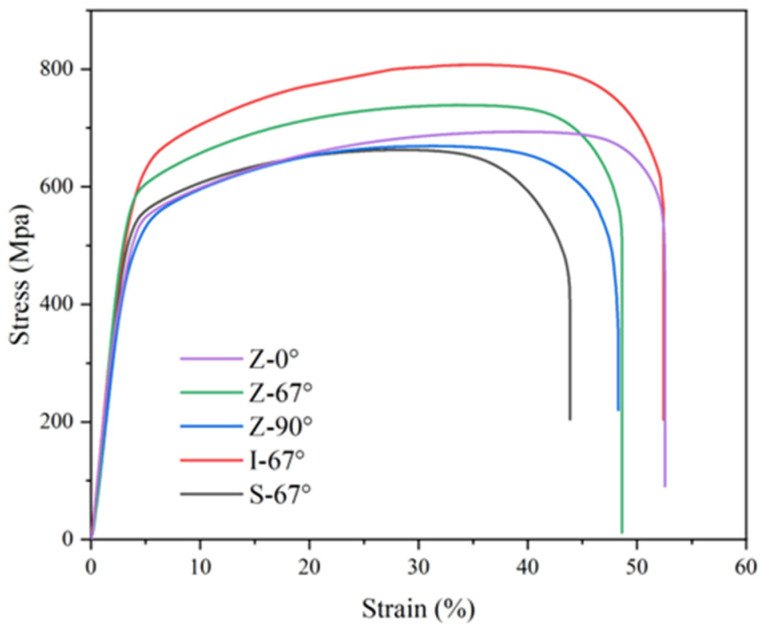
Stress–strain curves of SLMed-316L stainless steel.

Figure 13 shows the stress–strain curves of the manufactured 316L stainless steels by SLM, prepared with the same laser energy and different scanning strategies, as well as the tensile test of a forged sample. As can be seen from the figure, the 316L stainless steel prepared by SLM does not exhibit significant yield during the tensile stage.

In order to analyze the effect of the scanning strategy on the tensile properties of additively manufactured 316L stainless steel, the yield strength, tensile strength, and elongation of the specimen were obtained according to the stress–strain curve, and the results are shown in Table 3 (tensile properties). The elongation corresponding to the fracture of the specimen is taken as the elongation of the tensile test. In the experiment, the yield strength of the sample prepared by SLM is between 450 and ~550 MPa, the tensile strength is between 650 and ~810 MPa, and the elongation is between 43 and ~53%, whereas the yield strength of forged 316L stainless steel is 336.3 MPa, the tensile strength is 589.6 MPa, and the elongation is 43.3%. Compared to the worst additive specimen (Z-0°), the yield strength and tensile strength were reduced by 135.5 MPa and 73.4 MPa, respectively.

Combined with the stress–strain curve analysis of the specimen, the 316L stainless steel specimen prepared by SLM still exhibits good plasticity before reaching the tensile strength, and the final tensile strength value is also large. The reason for this is that the stainless-steel liquid in the SLM sample is rapidly cooled during the lamellar manufacturing process, and the grains in the ultra-high sample are refined to form a dense sub-grain structure. Using the metal Hal-Petch formula, the fine sub-grain structure can effectively improve the mechanical properties of the material, and the sub-grain structure of the sample can effectively prevent the propagation of tensile cracks. The yield strength and tensile strength of the specimen were improved, and the tensile properties were good [42]. By comparing the tensile test results of forged specimens and fabricated specimens, it can be seen that the 316L stainless steel prepared by serial energy density and scanning strategies in the experiment has better strength and slightly higher ductility than the forged specimens. The use of nitrogen as a shielding gas can increase the dislocation density in the sample and optimize the ductility of the sample [43].

**Table 3:** Tensile properties of the printed samples.

Sample	Z-0°	Z-67°	Z-90°	S-67°	I-67°	ISO	S.J. [10]	C.P. [44]
YS (MPa)	471.7	512.4	457.0	498.1	533.0	≥205	511.6	569
UTS (MPa)	663.0	739.1	669.6	693.7	807.7	≥515	621.7	667
E (%)	43.9	48.6	48.3	52.5	52.4	≥35	20.4	33

The I-67° specimen had the best tensile performance, with a yield strength of 533.0 MPa, a tensile strength of 807.7 MPa, and an elongation of 52.4%. The yield strength of the S-67° specimen of the strip laser path was 498.1 MPa, the tensile strength was 693.7 MPa, and the elongation was 52.5%. As can be seen from Figure 10 and Table 1, the elongation of the I-67° island laser path and the S-67° strip laser path samples are significantly higher than that of the overall Z-shaped laser path, which is increased by 9.1% and 9.2%, respectively; however, the yield strength of 316L stainless steel prepared by the three laser paths is relatively similar. The difference between the maximum and minimum values is only 20.6 MPa. Therefore, the existing laser remelting lap area in the samples prepared by island and strip laser paths improves the ductility of the samples to a certain extent and optimizes the tensile properties of the samples.

**Figure 14:**
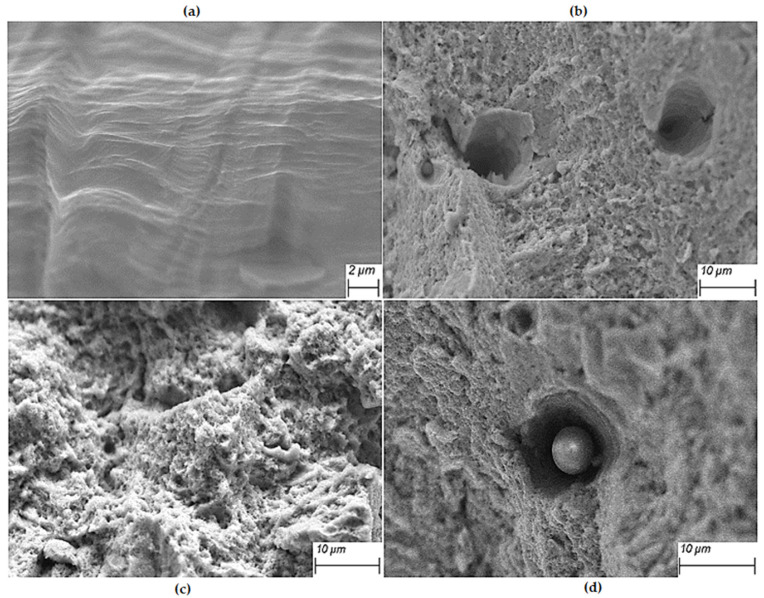
Tensile fracture morphology of SLM specimen with Z-type laser path: (**a**) lower part of Z-0°; (**b**) upper part of Z-0°; (**c**) Z-67°; (**d**) Z-90°.

Figure 14a,b shows the fracture morphology of the Z-0° specimen with an overall Z-shaped interlaminar rotation of 0°. Figure 14a shows the fracture morphology of the Z-0° specimen near the matrix side, where the specimen is formed under good heat dissipation conditions and a large temperature gradient, and the scanning laser path of the 0° interlayer rotation is the same. The specimen will grow along the direction of the existing grains, and the crack source will form and diffuse at the grain boundaries under the action of tensile forces, resulting in the crack shown in Figure 14a. In the upper part, away from the substrate, the heat dissipation conditions of the specimen are poor, and the microcracks that accumulate in layers cannot be formed. Figure 14c shows the fracture topography of the Z-67° specimen with a 67° rotation across the entire Z-beam laser path and interlayers. No obvious defects were found at the fracture of the specimen, and the fracture distribution of the specimen was small (less than 1 micron). Figure 14d shows the fracture topography of the Z-90° specimen under the full Z-beam laser path and 90° interlayer rotation. The unmelted powder can be seen at the fracture, indicating that the defect at the port reduces the tensile properties of the specimen.

**Figure 15:**
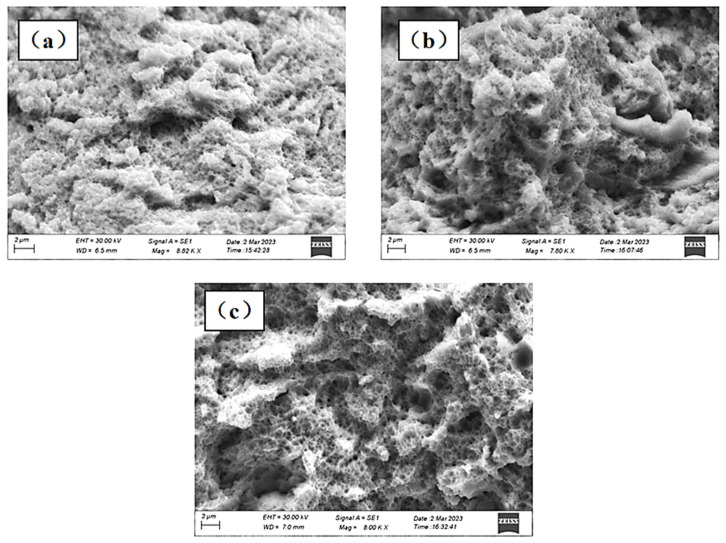
Tensile fracture morphology of 67° rotating samples and forged specimens between layers: (**a**) Z-67°; (**b**) S-67°; (**c**) I-67°.

Figure 15 shows the fracture topography of Z-67°, I-67°, S-67° specimens and forged specimens with 67° interlayer rotation. The fractures all presented a large number of undulating dimples in SLM 316L samples, which were typical ductile fracture characteristics consistent with good ductility [45]. Figure 15a shows the fragmentation of the entire Z-shaped laser path sample. It can be seen that there are many small dimples around the large dimples, and the small dimples are more uniform in size, and some brinks can be found at the bottom of the fine inclusions. Inclusions and second-phase particles often become the starting point for cracks, which increase and grow as the tensile force increases, eventually manufacturing a honeycomb at the fracture, causing the specimen to break. Figure 15b,c shows the fracture morphology of the S-67° and I-67° samples, respectively. Samples prepared by the island and bar laser paths contain fewer inclusions and are also composed of large and small size dimples compared to the Z-67° samples. The size of the small dimple is similar to the size of the sub-grain structure of the additively manufactured sample. The sub-grain structure of additive stainless steel is important for the tensile properties of the specimen.

## 4. Conclusions

In this study, 316L stainless steel was prepared by using three different scanning paths and three different interlayer rotation angles. The effects of different scanning strategies on the surface morphology, density, and corrosion resistance of 316L stainless steel were systematically studied, and the main conclusions are as follows:

(1) The 67° rotation between layers can effectively improve the manufacturing quality of stainless steel and help reduce the roughness accumulated layer by layer during the printing process. When the interlayer rotation is 0°, the laser path of each layer is exactly the same; the molding quality is poor, and the roughness is high;

(2) The overlapping part of the partition scanning is an example of good metallurgical bonding. The single laser path is short, and the by-products produced at a single time are few. The reliability of the molded specimen is low, and the molding quality is high. Samples prepared using the I-67° scanning strategy have the best molding quality;

(3) When the interlayer scanning paths are the same, the grains in the molten pool mainly grow in two directions perpendicular to each other. When the interlayer rotation is 90°, the grains are more likely to cross the melt pool boundary and produce epitaxial growth. When the interlayer rotates at 67°, the grain growth direction is more complex;

(4) In the scanning strategy used in the experiment, the sample with a 67° rotation between the layers showed good corrosion resistance, followed by a 90° rotation between the layers and a minimum of 0° rotation between layers;

(5) The specimens prepared by SLM have excellent tensile properties. The fracture morphology analysis of the specimen showed that there was an obvious ductile fracture at the fracture of the SLM specimen, and the size of the small dimples was similar to the size of the sub-crystalline structure, indicating that the good tensile properties of the additive manufacturing specimen were related to its sub-grain structure.

## Figures and Tables

**Table 1:** Chemical compositions (wt.%) of 316L ASSs investigated in this study.

Material	C	Cr	Ni	Mo	Mn	Si	P	S	O	Fe
Powder	0.008	16.73	10.95	2.62	0.88	0.56	0.01	0.004	0.052	Bal.

**Table 2:** Fitting parameters of SLM316L using the equivalent circuit.

Samples	Rs (Ω)	Rf (Ω)	Rct (Ω)	CPE_1_		CPE_2_	
				Q_1_ (Ω·s^n^)	n_1_	Q_2_ (Ω·s^n^)	n_2_
Wrought	15.14	4.6 × 10^2^	4.28 × 10^4^	5.19 × 10^−5^	0.82	1.03 × 10^−7^	0.66
Z-0°	19.16	4.11 × 10^4^	2.70 × 10^4^	4.6 × 10^−5^	0.82	6.7 × 10^−6^	0.78
Z-67°	21.37	6.68 × 10^4^	1.46 × 10^5^	1.69 × 10^−5^	0.91	4.32 × 10^−6^	0.75
Z-90°	18.37	1.55 × 10^4^	1.37 × 10^5^	1.6 × 10^−5^	0.90	3.5 × 10^−6^	0.64
S-67°	20.06	3.44 × 10^4^	2.44 × 10^5^	1.36 × 10^−5^	0.91	7.76 × 10^−6^	0.78
I-67°	20.1	7.01 × 10^4^	2.25 × 10^5^	1.68 × 10^−5^	0.92	3.82 × 10^−6^	0.72

## Data Availability

Due to the need for follow-up research, it is not convenient to disclose the data for the time being.
